# Primary care staff and health policymakers’ perspectives on the Israeli special team management program for complex patients and its further enhancement: a qualitative study

**DOI:** 10.1186/s13584-026-00792-x

**Published:** 2026-11-09

**Authors:** Vered Mintzer, Shai Ashkenazi, Eugene Merzon, Ayala Blau

**Affiliations:** 1 Adelson School of Medicine, Ariel University, Ariel, 40700 Israel; 2 Leumit Health Services, Tel Aviv-Yafo, 64738 Israel; 3 Department of Nursing, Faculty of Health Sciences, Ariel University, Ariel, 40700 Israel

**Keywords:** Health policy, Health economics, Complex patients, Chronic diseases, Teamwork

## Abstract

**Background/aim:**

The healthcare of complex patients is challenging, requiring holistic multidisciplinary teamwork. The targeted Team Management for Complex Patients Program (TMCP) was therefore initiated in Israel and implemented by Leumit Health Services. The aim of the current study was to examine the perceptions and insights of healthcare stakeholders regarding the program implementation and its further enhancement.

**Methods:**

A descriptive qualitative study using semi-structured remote videoconference interviews was conducted. A purposive sampling of primary care practitioners, managers and policymakers, with operational experience in the TMCP, was employed. Interviewees were categorized into three levels: field staff and service providers, mid-level management staff and high-level policymakers.

**Results:**

Five main themes were examined amongst the 22 interviewees: detection of complex patients for inclusion in the TMCP; quality and access to healthcare; team function, governance and accountability; and provider experience. These themes were extracted from the three levels of interviewees, all of whom endorsed a broad definition of complexity that integrates clinical, functional, mental and social dimensions and emphasized the critical role of multidisciplinary teamwork in complex patient care. Optimal patient identification combines clinical assessment (“bottom-up”) with data-driven lists (“top-down”). There was unanimous rejection of using age ≥ 65 years as a threshold, as complexity exists across all age groups. A consensus emerged on transitioning to personalized, goal-oriented care that integrates pharmacological and mental health consultations. The nurse was identified as the central coordinator for care integration, while the physician remained the primary clinical authority. Significant barriers mentioned by the interviewees included administrative focus on economic efficiency versus the frontline need for dedicated time and training. Participants preferred to use existing management structures rather than create new roles to maintain continuity of care within primary clinics, and unanimously supported program monitoring to track clinical outcomes.

**Conclusion:**

Stakeholders participating in the study highlighted diverse insights and recommendations regarding the healthcare policy for managing complex patients. Effective teamwork by empowered and trained teams, patient-centered goals and rigorous monitoring seem fundamental for the success of the Israeli TMCP.

**Supplementary Information:**

The online version contains supplementary material available at https://doi.org/10.1186/s13584-026-00792-x.

## Introduction

### The characterization of complex patients

With the increasing survival of patients with chronic diseases in the advanced modern medicine and the global aging population, the rates of complex patients and their burden are busting [1]. The precise identification of complex patients is fundamental to establishing appropriate health management policy [2, 3]. While patients are typically characterized by multimorbidity – including chronic conditions such as diabetes, hypertension, and respiratory, cardiac, or renal diseases – their complexity extends beyond clinical diagnoses [4–6]. It encompasses psychological, cognitive, functional, and social determinants, as well as poor self-management, all of which pose significant challenges to achieving optimal clinical outcomes [7–9]. A recent study demonstrated that clinical markers of complexity include polypharmacy, abnormal laboratory indicators, difficult-to-manage comorbidities, and high healthcare utilization, such as frequent emergency department (ED) visits and hospital admissions [3].

The INTERMED complexity assessment grid, based on four domains – biological, psychological, social, and health system involvement – was developed to evaluate biopsychosocial complexity in primary care [2]. Although empirically validated, this tool presents significant logistical challenges for practical implementation in clinical settings. Nevertheless, the gold standard for identifying a patient as complex is the clinical judgment of primary care physicians (PCPs), which leverages their holistic perspective on patients’ multifaceted medical complexities and care requirements [3, 4, 6].

### Management of complex patients

Managing complex patients is challenging and a high-cost endeavor that strains public health systems [5, 10–12]. While the PCP is viewed as the central case manager [2, 5, 12, 13, 14], effective healthcare requires a coordinated, multidisciplinary effort [9, 15–17]. Evidence suggests that nurses’ involvement in enhancing communication among healthcare providers yields significant positive outcomes [18–20].

There is a growing consensus that care must shift toward a patient-centered, holistic model that integrates mental health and social determinants of health alongside clinical parameters [7, 21, 22]. Indeed, relying solely on rigid disease guidelines often fragments care and increases the risk of adverse outcomes [23, 24]. To mitigate these issues, modern health policies prioritize defragmentation [23] and multidisciplinary collaboration [17, 25] to deliver measurable benefits [26–29].

### The Israeli team management for complex patients program

The Team Management for Complex Patients Program (TMCP) is a proactive, multidisciplinary initiative of the Israeli Ministry of Health (IMoH) [30]. It primarily targets complex patients aged ≥ 65 years characterized by multimorbidity – defined as having ≥ 2 chronic conditions – who are managed in the ambulatory setting, are not homebound and do not require palliative care, but are characterized by clinical, mental, cognitive, functional, social, or behavioral complexity that necessitates active involvement of a care team to prevent health deterioration. Patients who participated in the program are identified by their PCP as presenting formidable barriers to effective care delivery by traditional disease-management protocols. The TMCP operates in ambulatory settings and provides individualized, coordinated interventions through an interdisciplinary team (see Supplementary Material 1 for a full description).

#### Major required components of the TMCP are

- A core TMCP team should be established, which includes the PCP (case manager), nurse, social worker, and administrative care coordinator. Care should be proactive: the program required recurrent core team contacts, including at least 1 physician encounter/quarter, 2 nurse encounters/quarter, 1 administrative coordinator encounter/quarter and 1 social worker encounter/6 months. During nursing encounters, nutritional assessment, depression screening, pain assessment, diabetes monitoring, functional assessment, and medication-administration education are required.
- Within the first month after enrollment, the patient should undergo assessment by the PCP and nurse and be contacted by an administrative care coordinator.
- A personalized care plan should be prepared within 2 months of enrollment, jointly in a proactive meeting between the care team and the patient. The care plan should integrate the findings of the multidisciplinary assessment and define patient-specific clinical and care-management priorities, including personal goals.
- According to individual patient needs, the care plan could incorporate nutritional status, medication and pharmacotherapy reviews, functional and psychosocial support, and other clinically required issues.
- The team coordinator facilitates implementation of the care plan, including appointments, consultations and assistance in overcoming administrative barriers.
- Relevant electronic medical records should be accessible to members of the multidisciplinary care team.
- Within the first 3 months, an assessment by a social worker is required.

The program is benchmarked against strict IMoH performance-based support criteria, a national funding framework designed to promote multidisciplinary care for severely complex patients [30]. Under this framework, participating HMOs are required to implement predefined components of structured care and to document their delivery (details in Supplementary Material 2).

### Implementation of the TMCP in leumit health services

The TMCP was implemented in 2020 in Leumit Health Services (LHS), a nationwide Israeli Health Maintenance Organization (HMO) serving a diverse population of over 725,000 members. To ensure clinical consistency, PCPs underwent training via a dedicated instructional model focused on the program’s core definitions. Notably, final enrollment remains at the clinical discretion of the PCPs, leveraging their longitudinal expertise to identify a subset of highly complex individuals who would benefit from the TMCP.

Although this relatively novel Israeli program has already been operated in LHS for a few years, broad insights into the perspectives of caregivers, health managers and policymakers regarding its optimal implementation and potential improvements are not available.

## Materials and methods

### Aims of the study

This qualitative study aimed to explore the perceptions of a diverse range of Israeli health stakeholders regarding the implementation and further improvement of the TMCP, including field primary care staff (PCPs, nurses, social workers, and care coordinators), medical and nursing managers and healthcare policymakers. Exploring these perspectives is important for the successful ongoing implementation of the program within the Israeli national healthcare policy, and its further enhancement.

### Study design

The current research employed a descriptive qualitative methodology to examine the opinions of diverse healthcare stakeholders regarding the TMCP. Data collection involved semi-structured remote videoconference interviews, informed by the INTERMED complexity assessment tool [6] and the Teamwork Effectiveness Assessment Module (TEAM) for primary care [28].

### Recruitment and study population

**Fig. 1:**
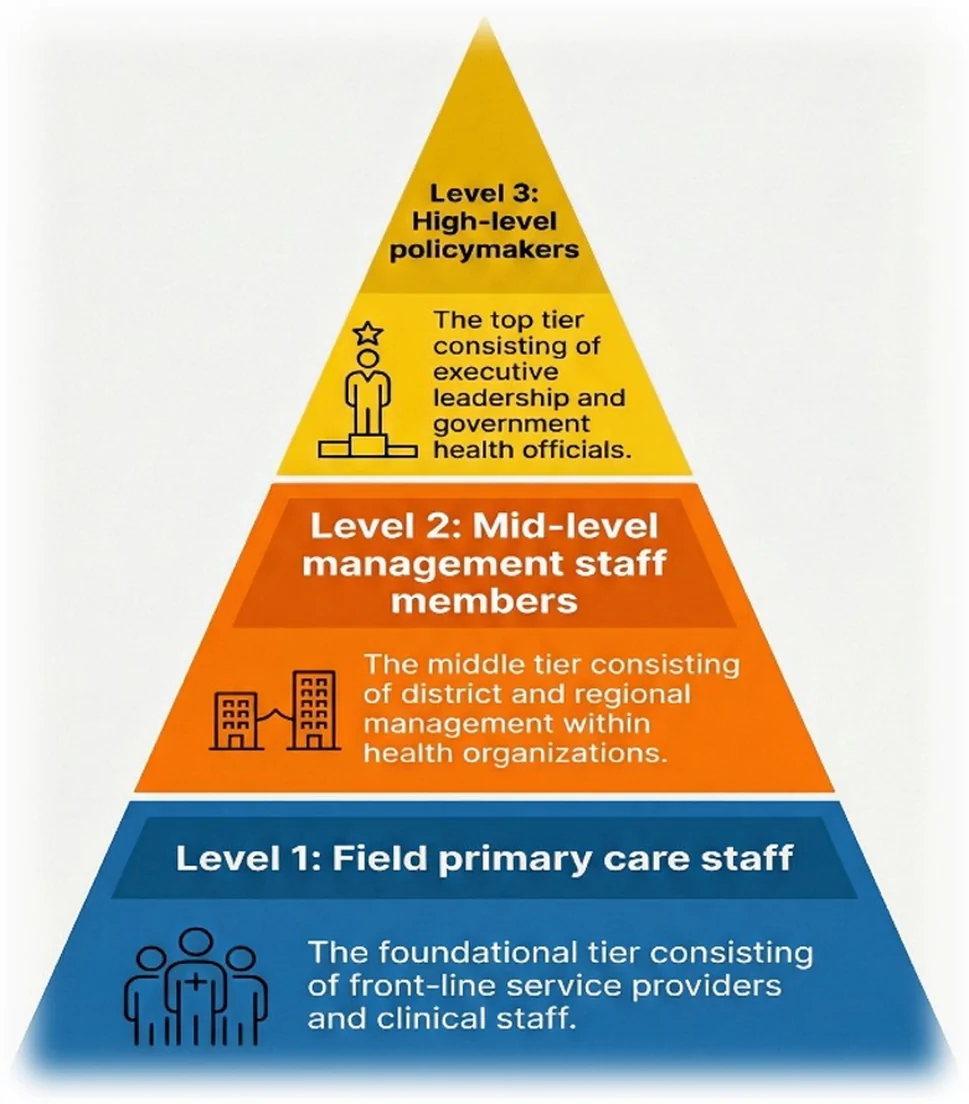
Three levels of healthcare stakeholders of the interviewees: field primary care staff and service providers (group 1); mid-level health management staff (group 2); and high-level policymakers (group 3)

A purposive sampling strategy was utilized to identify and recruit key informants for this study and to ensure diverse professional representation. The sample included individuals associated with the TMCP, such as nurses, PCPs, social workers, care coordinators, medical and nursing managers, and policymakers. We categorized interviewees into three levels, as shown in Fig. 1. The researchers determined the appropriate interviewees to cover all districts (North, South, Central, and Jerusalem), and national-level policymakers, based on prior familiarity and through regional and district nursing or medical managers. The lead researcher (VM) contacted potential interviewees directly via email to obtain their consent and schedule an interview date.

The final sample size was dictated by data saturation, namely the point at which further interviews yielded no additional novel insights [31]. Specifically, the recruitment aimed to balance the number of participants among nurses, PCPs, regional/district medical managers, and regional/district nursing managers. Furthermore, participants were selected from diverse geographic locations nationwide, including both central and peripheral areas, to ensure comprehensive data that reflect all spatial contexts across the country. The number of individuals interviewed by job title and level in the healthcare system is summarized in Table 1.

**Table 1:** The number of individuals interviewed by job title and level in the healthcare system

Pyramid level	Job title/role (number of interviewees)
**Base Layer - Group 1**	Nurses (3)
Field primary care staff	PCPs (2)Social workers (2)Care coordinators (2)
**Middle Layer - Group 2**	District or regional nursing managers in HMO (4)
Mid-level management staff	District or regional medical managers in HMO (3)
**Top Layer -Group 3**	Chief HMO Nursing Director (1), HMO Medical Director
High-level policymakers	(1), HMO CEO (1), Director of Primary Care and Quality Improvement in HMO Department of Medicine (1), Head of the Community Health Division in IMoH (1), Senior Manager of Clinical Nursing Specialties in IMoH (1)

*CEO, Chief Executive Officer; HMO, Health Maintenance Organization; IMoH, Israeli Ministry of Health

### Research process

Data was collected over a 13-month period, from December 1st, 2024 to December 31st, 2025. A single researcher (VM), a registered nurse with patient affiliation as head clinic nurse at LHS and experience in the TMCP, conducted all participant interviews remotely (via the Zoom platform), which typically lasted 30–60 min. Interviews were based on a semi-structured guide that was prompted during the interviews. The questionnaire also included open-ended questions to explore the established criteria and elicit participants’ personal views; the completed interview questions and schedule are available in Supplementary Material 3. To enhance reliability, the interviewer summarized the key points and invited the interviewee to provide further clarifications or amendments. Following recording and verbatim transcription, the data were subjected to thematic analysis. This qualitative process followed an iterative approach: collecting, coding, and content analysis of data concurrently [32, 33]. To ensure rigor, transparency and comprehensive documentation, reporting adhered to the COREQ (Consolidated Criteria for Reporting Qualitative Research) guidelines [34].

### Data analysis

**Fig. 2:**
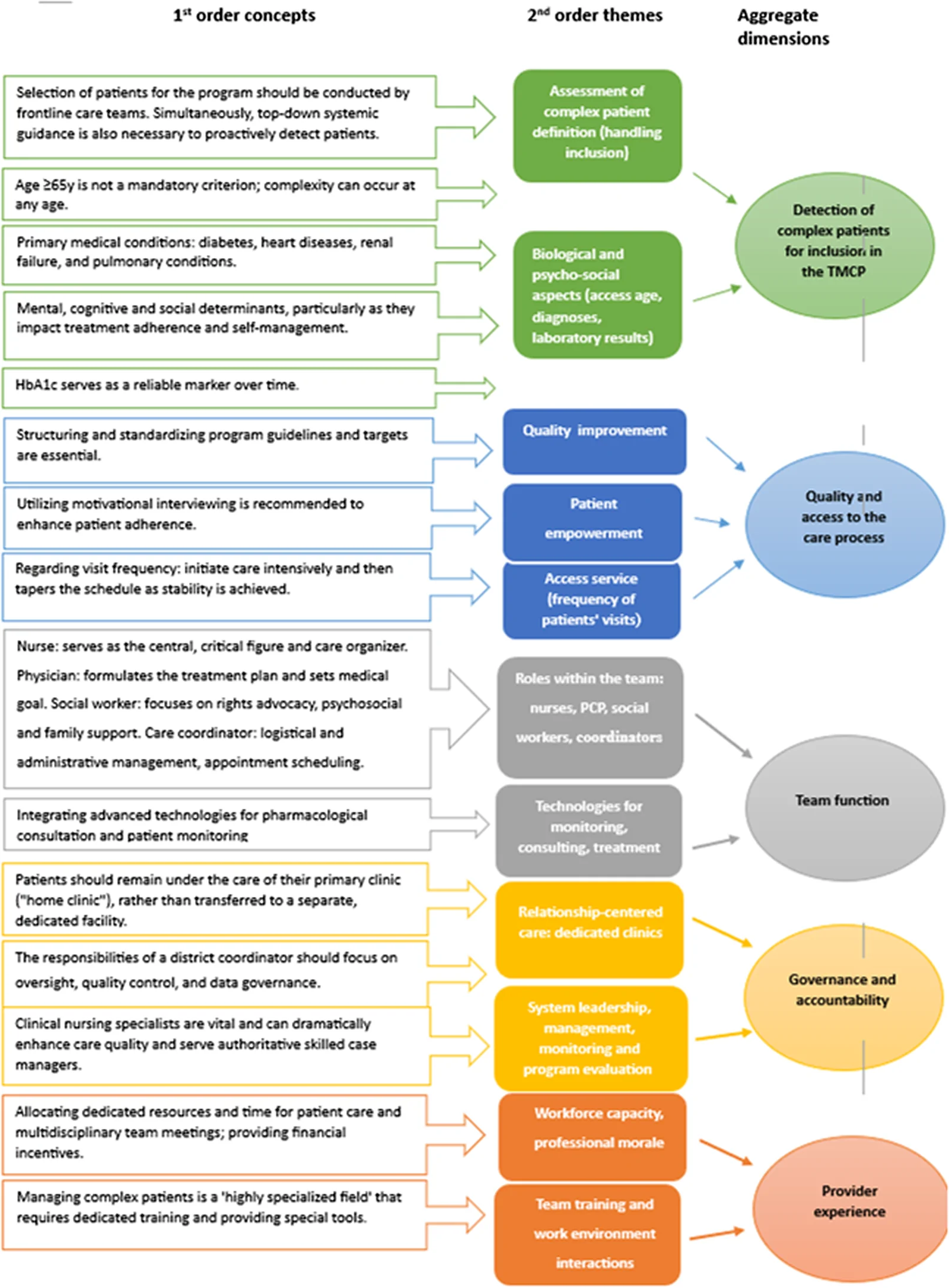
Data structure illustrating the analysis process

Data were analyzed using a descriptive qualitative approach grounded in thematic analysis [35]. In the first stage, the primary researcher conducted the interviews and recorded and transcribed them. Audio recording and verbatim transcription enabled close adherence to the source data, allowing an in-depth understanding of participants’ narrative meanings and tracking the internal logic of each narrative [33]. In the second stage, an initial analysis of the interviews was conducted, encompassing an individual reading of each transcript, preliminary content analysis, and an initial mapping of emerging content against existing conceptual models, namely the INTERMED complexity assessment tool and the TEAM framework [6, 28]. The third stage involved open coding, through a collective analysis of all transcripts and categorization into topics guided by the interview schedule. An analytical examination was conducted by categorizing and differentiating among emerging contents, labeling identified topics, aggregating them into overarching categories, and sorting sub-themes within each category. This process was initially performed independently and subsequently in collaboration with an additional researcher (AB). As the participants were classified into three levels (Fig. 1), interview summaries were generated for each group using NotebookLM (Google AI) to facilitate initial data organization. All AI-generated summaries were subsequently reviewed and verified by the researcher to ensure accuracy and contextual integrity. In the fourth stage, the content within each topic was refined through an iterative process that involved repeatedly reanalyzing text segments to ensure appropriate thematic allocation. In the fifth stage, axial coding was conducted to unify thematic categories from initial codes and synthesize recurring themes. In the sixth stage, distinctions were established among sub-themes within each overarching category, followed by aggregation and broad theoretical conceptualization of the overarching categories [36]. Figure 2 presents the data structure conceptualizing the analytical approach (based on [37]).

### Ethical aspects

Following ethical approval of LHS (LEU-0013-24), oral informed consent was obtained from all participants, and their privacy was strictly protected through anonymity and confidentiality. Furthermore, participants appreciated their right to withdraw from the interview at any stage without penalty. Interview sessions were recorded only with the participants’ explicit consent to audio recording. All acquired data were kept confidential and secured within a password-protected computing environment.

### Rigor

A robust framework to ensure trustworthiness across four key dimensions was adopted: credibility, transferability, dependability, and confirmability [38]. By utilizing triangulation, rich descriptions and rigorous documentation, the research achieved high levels of transparency. Additionally, reflexive journaling and peer debriefing supported the confirmability of the data. These collective efforts ensure that the findings are not only precise and reliable but also highly useful for future academic inquiry and practical implementation.

## Results

### Study participants

Of the 25 healthcare stakeholders invited to participate in this study, three dropped out for personal reasons. Participants were employed across broad Israeli geographic regions and in various sectors, including nursing, medicine, social work, administration, and IMoH management. They occupied various hierarchical levels, ranging from frontline field staff to district and regional managers, and division and sector heads. The participants had extensive professional experience and seniority in their professions, with 45% having > 30 years in their roles. Table 2 presents the demographic characteristics of the participants.

**Table 2:** Demographic characteristics of the study participants

Variable		Number (*n* = 22) (%)
Gender	Male	10 (45.45%)
	Female	12 (54.54%)
Age, years	31–40	3 (13.63%)
	41–50	3 (13.63%)
	51–60	7 (31.81%)
	61–70	2 (10%)
	*missing*	*7 (31.81%)*
Education	M.D.	8 (36.36%)
	R.N.	7 (31.81%)
	PhD. R.N.	2 (9.09%)
	Other B.A	5 (22.72%)
Department	Nursing	8 (36.36%)
	Medicine	7 (31.81%)
	Ministry of Health	2 (10%)
	Social Work	2 (10%)
	Administration	3 (13.63%)
Seniority, years	1–10	6 (27.27%)
	11–20	4 (18.18%)
	21–30	2 (10%)
	31–40	10 (45.45%)
Work District	Jerusalem	4 (18.18%)
	Center	5 (22.72%)
	North	3 (13.63%)
	South	1 (4.54%)
	National	9 (40.9%)

### Interviews’ analysis

The interviews’ analysis is summarized in five main categories, which were developed through analysis (Fig. 2): Detection of complex patients for inclusion; Quality and the care process; Team function, governance and accountability; and Provider experience.

#### Detection of complex patients

##### Assessment of complex patient definition

All groups endorsed a broad definition of complexity that integrates clinical, functional, mental, and social dimensions. Group 3 emphasized that complexity involves also financial barriers and psychosocial stressors. All agreed that the final decision on inclusion must prioritize the patient’s willingness to cooperate and adherence to treatment. *“The definition is too general and fails to address the gap between the patient’s medical needs and his ability to implement the medical treatment; it is precisely this gap that makes a patient ‘complex’ “(social worker 14).* There was strong consensus that the selection process for patients should not be limited to PCPs; nurses and social workers should also be consulted, as they possess deep knowledge of patients’ home environments and family dynamics. *“The idea that only a physician can select the patient for the program is a ‘significant shortcoming*,*’ as the nurse is much more familiar with the patient’s functional and environmental conduct at home“(nurse 12).*

The majority supported a “hybrid model” for patient recruitment. This approach combines top-down, data-driven lists (to identify “under the radar” patients) with bottom-up, clinically-based decisions. While data provide the foundation, the PCP and nurse’s discretion is paramount in assessing the patient’s dynamic environment and potential for cooperation.

The program prioritizes patients with modifiable clinical, psychiatric, or self-management challenges for which systemic intervention can have a measurable impact, while generally excluding end-of-life cases. The primary physician’s ability to cope with the patient’s challenge was important. *“A patient should be defined as ‘complex’ when*,* after investigating the causes of his imbalance*,* it becomes clear that the primary physician cannot handle the case solo. The doctor recognizes that external support is mandatory for successful treatment” (chief medical officer 21)*. A unique insight emerged regarding the “physician factor”: *“complexity is often defined by the interaction between a patient’s needs and the physician’s specific capacity (experience*,* workload*,* attitude/personality*,* and expertise) to manage the case” (medical manager 17).* Table 3 illustrates comparisons among the groups regarding the assessment of the complex patient definition theme.

**Table 3:** The assessment of the complex patient definition theme by the three healthcare groups

Theme	Group 1	Group 2	Group 3
Assessment of complex patient definition	Involving multiple healthcare professionals in the patient selection process is emphasized, alongside a focus on the discrepancy between patients’ needs and their capacity to carry out treatment. Furthermore, ‘treatment adherence’ and the patient’s explicit ‘consent and willingness to participate’ are critical inclusion criteria, along with polypharmacy and frequent hospital admissions.	Criteria reflecting the burden on the healthcare system should be incorporated in the inclusion process, such as the frequency of emergency department visits and hospitalizations. Polypharmacy and major medical complications (such as dialysis dependency) should be explicitly addressed. Emphasis must be placed on absence of familial or environmental support, and presence of psychiatric conditions (e.g., depression) that contribute to self-neglect or hinder effective healthcare.	Emphasizing that the definition of complexity also depends on the dynamic interaction between the patient and his PCP, including the physician’s capacity to manage the case. Physician characteristics (e.g., age, professional specialty, workload, and clinical perspective) ought to be integrated into the equation. Frequent emergency department visits and hospitalizations reflect healthcare system utilization and indicate breakdowns in community-based management.

##### Biological and psycho-social aspects

Diabetes and cardiac diseases/heart failure were the leading diagnoses in complex patients (mentioned by 13 participants), followed by vascular diseases, stroke, renal failure, and pulmonary diseases. There was an emphasis on diagnoses that require a high level of self-management or those that patients tend to neglect. Autoimmune diseases were repeatedly mentioned as related to complicated pain management, leading to referrals to pain clinics and frequent hospitalizations. Poor mental health, depression, or psychiatric disorders were highlighted as significantly impacting patient adherence, leading to self-neglect and frequent ED visits (12 participants). Lastly, cognitive impairment/dementia was noted for its impact on overall care management (8 mentions).

While groups 1 and 2 stressed key laboratory markers for identifying complex patients (including serum albumin, renal function and inflammatory indicators [such as C-reactive protein]), group 3 warned against “treating the lab results” rather than the individual. There was unanimous rejection of using age 65 years as a rigid threshold, as complexity is seen across all ages (e.g., young diabetic patients, trauma survivors). All participants agreed that extreme healthcare utilization may signal complexity, but mentioned that the broader issue of resource utilization was an extensive field that must be thoroughly examined. *“I would actually focus on patients who have few physical visits but high engagement via digital communication… that is likely where there is still potential” (chief medical manager 19).*

#### Quality and access to the care process

Table 4 outlines comparisons among the groups aligned with the quality assessment theme.

**Table 4:** The perceptions of the quality improvement theme by the three healthcare groups

Theme	Group 1	Group 2	Group 3
Quality improvement	Consensus on using personalized treatment plans that prioritize high accessibility, pharmacological optimization, and removal of bureaucratic barriers, with personalized goals. Additional key objectives: physical activity, smoking cessation, and treating sleep disorders.	Advocated a multidisciplinary baseline assessment, with emphasis on pharmacological consultation. Stressed the need to focus on managing depression as a major barrier to adherence, and preventing falls.	Encouraged standardizing program guidelines and metrics (including targets and outcomes) to facilitate future program evaluation, while maintaining clinical flexibility.

All participants agreed that treatment plan objectives should be patient-specific, addressing clinical, social, psychosocial, and pain management needs. There was broad consensus in group 1 that successful implementation requires specialized staff training, technological support (e.g., short educational or tutorial videos and SMS reminders), and potentially financial incentives to boost adherence. Additionally, streamlining bureaucratic and technical services (e.g., scheduling assistance) and maintaining high availability while providing a personal touch (e.g., proactive phone calls or personal contact details). However, it was stated that *“In order to increase program adherence*,* it is crucial to ensure that we are not exhausting the patient. Staff members must be provided with tools to enhance the patient’s motivation for treatment. This could be an additional component of the staff’s medical training” (chief manager 19).*

Recommended frequencies of scheduled contact included: quarterly physician reviews; twice quarterly to monthly nursing follow-ups, depending on the patient’s stability; at least monthly social worker/care coordinator contacts; and a quarterly staff meeting. The need to allocate appropriate dedicated timing for these complex patients was highlighted: *“The solution is to allocate specific time for these patients. PCPs are granted extended sessions for complex cases rather than the standard ten minutes. It is purely a managerial decision to allot more time to doctors for complex patients” (chief medical manager 21).*

Participants across all groups perceived the program as highly successful in improving diabetes control, treatment adherence, and functional status. Most clinicians rejected automatic discharge from the program, suggesting a minimum follow-up period of a year with reassessment every 6–12 months. Transitioning out of the program was contingent upon achieving clinical stability, meeting predefined goals, and demonstrating patient self-responsibility. A minority view complexity as a permanent state, suggesting these patients should remain under constant supervision unless they refuse to cooperate.

#### Team function

All participants emphasized the crucial importance of multidisciplinary teamwork for the program’s success, with a core team comprising a physician, nurse, social worker, and coordinator. All groups agreed that the nurse should be identified as the most effective ‘case manager’ for daily operations, providing essential logistical support, ensuring high availability and seamless navigation through the healthcare system, and taking responsibility for patient education. There was a clear consensus that the PCP is the ‘medical authority’ who sets medical goals, management plans, medications and referrals. However, four respondents noted that the choice of the program leader ultimately depends on individual professional capabilities rather than the role alone. Group 2 emphasized the need for specialized training for team leaders. The social workers emphasized the rights advocacy and emotional support.

There was broad support for integrating technologies, including virtual consultations, remote monitoring, and clinical dashboards, as essential tools for optimizing patient follow-up. A critical requirement was to provide these tools fully embedded within the existing electronic medical records to avoid administrative overload. Participants rated this as a top priority, with experts emphasizing its potential to streamline care management and enhance clinical outcomes.

#### Governance and accountability

Most participants supported keeping the care of complex patients within the TMCP in primary clinics, to ensure continuity and accessibility. Some suggested that mobile expert teams should support ‘weak’ clinics or provide virtual regional consultations: *“The care team can be regionally based; a team can be responsible for a cluster of clinics within a specific area*,* dividing its time among them” (Chief manager 21).* Table 5 summarizes the perceptions of system leadership, management and monitoring of the program theme by the healthcare groups.

**Table 5:** The perceptions of system leadership, management, monitoring and evaluation of the program theme by the three healthcare groups

Theme	Groups 1 and 2	Group 3
System leadership, management and monitoring of the program	Complex care management is a distinct field requiring specialized clinical training to serve authoritative, highly skilled case managers. Establishing a formal nurse practitioner (NP) training for complex patients, and a ‘district nursing coordinator’ role, overseeing program execution and data management, are suggested.	Current implementation of the TMCP should rely on existing NPs, as specialized training is not yet a scalable requirement for the current TMCP. Group 3 was divided on adding management layers (a new role); some argue that the district nursing and medical managers should control the program.

#### Provider experience

##### Workforce capacity and professional morale, team training and work environment interactions

Participants in groups 1 and 2 repeatedly emphasized the importance of strengthening multidisciplinary teamwork, for example, by dedicating time to meetings. They also highlighted the importance of providing tools and training to clinical staff to enhance their competence regarding clinical, psychosocial, and functional complexities; this includes guidance on care implementation and strategies for patient communication aimed at increasing patients’ adherence. These groups also emphasized the need to empower the staff through incentives. Furthermore, any newly created role (such as case manager within a clinic) must be ‘formally standardized’ with a designated position and salary. Group 3 appreciated the need to allocate more time and resources for the participating staff but noted that this should not entail financial compensation.

## Discussion

### New insights and their relation to current literature

To the best of our knowledge, this is the first study to comprehensively evaluate the IMoH TMCP and its potential future enhancements, based on the perceptions of 22 diverse health stakeholders, using the TEAM framework [28]. The health stakeholders perceptions show that the definition of a complex patient is perceived as broad, encompassing clinical, functional, mental, and social components. Similarly, interviews with PCPs in Canada, the Netherlands, and the US found that social and behavioral factors play major roles in patients’ complexity [39–41]. The inclusion in the TMCP is fundamentally dependent on the primary physicians’ in-depth acquaintance with the patients, with important inputs of the nurses and social workers, in concert with a Dutch study to identify “high-need, high-cost, chronically ill patients“ [42]. The IMoH established age 65 years as the threshold for program eligibility, but this limit was not perceived by study participants, as previously advocated [8, 10, 43].

Study participants highlight that quality care should be personalized and tailored according to patients’ specific needs. This aligns with the recognized shift from disease-oriented guidelines to patient-centered, goal-oriented care. As documented in previous studies, such an approach is vital for complex patients because applying multiple, sometimes conflicting, disease-specific protocols is unlikely to succeed [16, 44–46]. Incorporating pharmacological consultations, especially for managing pain and depression, was highlighted by our interviewees as essential to improved health outcomes. Previous studies have indeed shown the importance of controlling depression [47, 48], emotional distress [21, 49] and of pharmacological consultations [20, 50, 51] in the management of complex patients. Care coordination assistance, proactive follow-up calls, reducing bureaucratic barriers, and providing individualized attention were reported as pivotal [52], as emphasized in our study.

The study documented some gaps in the perceptions of frontline health professionals and high-level policymakers regarding the execution of the IMoH TMCP. Field staff emphasized that successful implementation requires specialized staff training, technological support, potential financial incentives, dedicated time for enrolled complex patients, team meetings, and also suggested ‘district coordinator’ positions. High-level policymakers suggested that current implementation of the TMCP should rely on existing trained positions, leaving schedule prioritization and resource distribution to clinic-level decisions. Policymakers encouraged standardizing program guidelines and metrics (including targets and outcomes) to facilitate future program evaluation. It seems to us that the practical implications of these gaps are that close contact between primary care staff and policymakers, with special attention to specialized staff training, technological support, dedicated time and enabling team meetings – are crucial for the success on the program.

The ‘Minimizing Error, Maximizing Outcome’ (MEMO) study highlighted strong correlations between adverse structural factors, such as high workload and poor organizational culture, and clinicians’ burnout and diminished commitment to the profession [53]. It strongly emphasized the need to provide team members with specialized training to communicate with complex patients and understand their multifaceted needs [15, 54–57].

A broad consensus emerged that nurses typically possess the necessary skills to lead care coordination, given their orientation towards holistic patients’ needs, as was elaborated in previous Israeli qualitative studies and literature reviews [58–61]. However, participants consistently identified PCPs as the ultimate medical authority. Finally, there was a consensus on the need to oversee and monitor the new TMCP, to determine program outcomes and inform healthcare policy changes.

### Strengths and limitations

The strengths of qualitative methods include their ability to provide in-depth insights into the topic under study [59, 62]. The present qualitative framework, using diverse levels of healthcare stakeholders, enabled the identification of specific perceptions and innovative management approaches that a purely quantitative analysis might overlook.

A limitation of this study is the relatively small number of participants, which hampers especially comparisons among stakeholder subgroups (such as various healthcare professions, levels of policymakers, ages, years of experience, and geographical regions). In addition, because healthcare systems vary across HMOs and countries, the program and its implementation in other locations require adaptation, although the principles for treating complex patients are similar. A major limitation is that the study did not include interviews with patients and family caregivers. Their perspectives are essential for understanding barriers related to accessibility, continuity of care, self-management, emotional burden, and trust in the healthcare system.

### Health policy implications of the study results and conclusions

The results of the in-depth interviews with primary care practitioners, healthcare managers, and policymakers have essential implications for current and proposed future healthcare policies for complex patients, which are summarized below.

- The definition of complexity for the inclusion in the program should be broad, integrating clinical, functional, mental, social and financial dimensions, with no age restriction.
- Multidisciplinary teamwork is viewed as essential for the success of the program.
- The PCP is the ‘medical authority’ who sets medical goals for included patients; the nurse is viewed as the ideal “case manager” due to the holistic orientation and ability to identify diverse patient needs.
- A recurring theme across all stakeholder levels, especially in the field staff, was the need for structured and targeted training of healthcare professionals engaged in the TMCP in the management of complex patients.
- Advanced digital and technological tools should be implemented for the monitoring, coordination, follow-up, and comprehensive management of complex patients.
- The care of complex patients should essentially remain within HMO primary care clinics to preserve continuity, accessibility, long-term therapeutic relationships, and reduce healthcare fragmentation.
- Policymakers encouraged standardizing program guidelines and metrics (including targets and outcomes) to facilitate future program evaluation and possible changes.

In conclusion, given the escalating number of complex patients worldwide, healthcare systems are required to develop new multidisciplinary care models, digital support systems, and structured educational programs focused on complexity-oriented healthcare, such as the Israeli TMCP. The findings of this study may therefore be relevant to a wide range of healthcare policymakers, academic institutions, and professional organizations. Future studies should examine the TMCP regarding its implementation fidelity, healthcare stakeholders, patient and caregiver satisfaction, clinical outcomes, cost-effectiveness and sustainability.

## Supplementary Information

Below is the link to the electronic supplementary material.

Supplementary Material 1

Supplementary Material 2

Supplementary Material 3

## Data Availability

The datasets generated and/or analyzed during the current study are available from the corresponding author on reasonable request.

## Declarations

### Use of AI in manuscript preparation

The authors used NotebookLM (Google AI) for generating interview summaries for each interviewee’s group to facilitate initial data organization. All AI-generated summaries were subsequently reviewed and verified by the researcher to ensure accuracy and contextual integrity.

## Abbreviations

TMCP: Team management for complex patients program
IMoH: Israeli ministry of health
LHS: Leumit health services
HMO: Health maintenance organization
PCP: Primary care physician
CEO: Chief executive officer
