# Supplementary material for: Primary care staff and health policymakers’ perspectives on the Israeli special team management program for complex patients and its further enhancement: a qualitative study
Source: Isr J Health Policy Res. 2026 Nov 9;15:49. doi: 10.1186/s13584-026-00792-x (PMC13746559; doi:10.1186/s13584-026-00792-x)
Supplement: Supplementary file 1 [file 13584_2026_792_MOESM1_ESM.docx]

**TMCP Qualitative Study – Draft Methods**

*Detailed description of the intervention, care pathway, clinical governance, and program adherence*

Supplementary Material for Article 2 in the Leumit Health Services complex-patient research series.

Prepared for review and subsequent integration with the full Methods section.

# Methods

## Study Setting and the Team Management for Complex Patients Program

The Team Management for Complex Patients (TMCP) program is a structured, proactive, multidisciplinary care-management program implemented by Leumit Health Services (LHS), a nationwide Israeli health maintenance organization (HMO), in accordance with an Israeli Ministry of Health (MoH) Support Criteria, a national funding framework designed to promote multidisciplinary care for complex patients.

A complex Patient was defined as a patient treated in a clinical, ambulatory setting who is not homebound and does not require palliative care, and whom the Care Manager has determined is characterized by clinical, mental, cognitive, functional, social, or behavioral complexity that necessitates the involvement of a care team to prevent health deterioration. Such a determination may be based on measurable criteria established by the Health Fund for this purpose.

Under this framework, participating HMOs are required to implement predefined components of structured care and to document their delivery. The program is intended to supplement, rather than replace, routine primary care by providing a coordinated longitudinal care pathway for patients who's medical and care-management needs exceed those usually addressed through standard disease-specific care.

Eligibility and physician-driven selection of patients for TMCP have been described in the previous study [1] in this research series. In brief, patients with at least 2 chronic medical conditions who were considered by their primary care team to require intensified, coordinated management could be enrolled in the program. The date of enrollment was defined as the index date for the present outcomes study.

## Initial Assessment and Personalized Care Plan

Following enrollment, the patient entered a structured initiation phase. Within the first month after the index date, the patient was required to undergo assessment by the primary care physician and nurse, and contact was established by an administrative care coordinator. The coordinator facilitated implementation of the care plan, including appointment coordination and assistance in overcoming administrative barriers to recommended care. Within the first 3 months, assessment by a social worker was also required.

According to Ministry of Health requirements, a personalized care plan had to be determined within 2 months of enrollment jointly in a proactive meeting between the care team and the patient. The care plan integrated the findings of the multidisciplinary assessment and defined patient-specific clinical and care-management priorities. Consistent with Ministry of Health guidelines, the plan included personal goals, proactive intervention strategies, and arrangements for managing urgent medical situations. According to individual patient needs, the assessment and care plan could incorporate nutritional status, medication and pharmacotherapy review, functional and psychosocial needs, chronic disease monitoring, and other clinically relevant issues. Recommendations were documented in the electronic medical record and were accessible to members of the multidisciplinary care team.

## Core Multidisciplinary Team and Proactive Longitudinal Follow-up

The core TMCP team (the 'basic team') consisted of personal primary care physician (case manager), nurse, social worker, and administrative care coordinator. Care was proactive rather than dependent solely on patient-initiated encounters. During continued participation, the program required recurrent contact with the core team, including at least 1 physician encounter per quarter, 2 nurse encounters per quarter, at least 1 social worker encounter every 6 months, and contact by the administrative care coordinator at least once per quarter. The coordinator followed implementation of recommended actions and assisted with access to appointments and other components of care.

## Extended Multidisciplinary Care

When clinically indicated, the core team referred patients to additional health professionals and medical specialists. These could include, among others, dietitians, clinical pharmacists, diabetologists, cardiologists, pulmonologists, pain-management physicians, physiotherapists, occupational therapists, and other specialists according to the patient's individual needs. According to the Ministry of Health framework, an Expanded Care Team was defined as the core team supplemented by at least two additional healthcare professionals selected on the basis of the patient's clinical or psychosocial needs. Encounters with these additional team members were required to be documented, and ongoing participation in multidisciplinary care was monitored as part of program adherence requirements. The decision to involve additional professionals remained clinically driven and was not intended as a uniform requirement for all patients.

**Structured Nursing and Multidimensional Assessment**

Repeated nursing encounters provided an opportunity for systematic assessment beyond disease-specific monitoring. Depending on patient characteristics and clinical indications, nursing care included nutritional assessment, depression screening, pain assessment, diabetes monitoring, functional assessment, and medication-administration education. These process measures were captured in the LHS electronic medical record and were subsequently evaluated as indicators of the intensity and breadth of care delivered during the program.

## Multidisciplinary Case Review and Shared Clinical Decision-making

An essential component of TMCP was recurrent multidisciplinary case review. At least once per quarter, participating core teams were required to conduct documented team discussions of patients enrolled in the program. These meetings were used to review clinical progress, unresolved medical and psychosocial needs, implementation of previous recommendations, barriers to care, and the need to revise the individualized care plan. Complex clinical cases could be discussed jointly with program leadership, enabling shared clinical decision-making and escalation of care when required.

## Training, Teamwork, and Ongoing Professional Support

Implementation was accompanied by continuing professional support for participating clinicians and staff. Program leadership conducted meetings with physicians and other team members, including discussion of complex clinical cases and joint consideration of management strategies. Teams received training aimed at strengthening multidisciplinary teamwork, coordination, communication, and shared responsibility for longitudinal management of complex patients. These activities were intended to support consistent implementation of the program across participating primary care settings.

## Clinical Governance, Quality Assurance, and Organizational Oversight

TMCP incorporated multilevel clinical governance and continuous quality monitoring. Program implementation and documented performance were reviewed not only within the local primary care team but also by program leadership and organizational management. Oversight extended through the LHS Medical Division and regional/district management structures. Program leaders reviewed implementation with participating teams, discussed selected patients and clinical challenges, and monitored adherence to required program activities. This multilevel oversight was designed to identify gaps in implementation, reinforce required care processes, and promote continuity and coordination across the organization.

## Program Adherence and Definition of Continues Participating in the Program

Continued enrollment required active participation in the structured program and documentation of its required components. Patients for whom required encounters, multidisciplinary team discussions, or other mandatory program activities were not performed and documented could not remain classified as continuously participating in the program. Consequently, the present 2-year outcomes analysis represents patients who remained enrolled and completed 2 years of TMCP follow-up, rather than all patients ever enrolled at the index date. This distinction is important when interpreting the study findings, because the outcomes cohort reflects sustained exposure to the intervention and a degree of patient and care-team engagement. According to MoH program requirements, participation was expected to continue for at least three months unless terminated because of circumstances outside the health organization's control, including hospitalization

## Conceptualization of the Intervention for the Present Study and the Difference from Routine Primary Care for Patients with Multimorbidity

For the present study, TMCP was therefore conceptualized as a multicomponent organizational and clinical intervention rather than as a single consultation or referral. Its principal components were: (1) physician-driven identification and enrollment; (2) timely multidisciplinary assessment; (3) creation of an individualized care plan; (4) scheduled proactive longitudinal follow-up by a defined core team; (5) needs-based access to an extended multidisciplinary network; (6) structured nursing and functional/psychosocial assessments; (7) recurrent multidisciplinary case review and shared decision-making; (8) professional training and teamwork support; and (9) multilevel clinical governance and continuous quality monitoring.

Reference

1. Mintzer V, Merzon E, Israel A, Ashkenazi S, Blau A, Magen E, et al. Clinical Variables Associated with Physician-Driven Inclusion in a Special Management Program for Complex Patients. J Clin Med. 2025;15:202. https://doi.org/10.3390/jcm15010202
