# Supplementary material for: Primary care staff and health policymakers’ perspectives on the Israeli special team management program for complex patients and its further enhancement: a qualitative study
Source: Isr J Health Policy Res. 2026 Nov 9;15:49. doi: 10.1186/s13584-026-00792-x (PMC13746559; doi:10.1186/s13584-026-00792-x)
Supplement: Supplementary file 2 [file 13584_2026_792_MOESM2_ESM.docx]

**Criteria for the Distribution of Support Funds by the Ministry of Health to Health Funds (HMOs) for the 2022 Support Year, pursuant to the Budget Foundations Law, 1985.**

**Target Population:** Support shall be granted for the **"Elderly Population,"** defined as: insured individuals aged 65 and older; patients with COPD (Chronic Obstructive Pulmonary Disease) or Heart Failure aged 45 and older; and dialysis patients of any age.

**Support Domain E - Implementation of a Team-Based Program for the Care of Complex Patients.**

**A. Definitions.** For the purposes of this support domain:

- **"Complex Patient"** – A patient treated in a clinical (ambulatory) setting who is not homebound and does not require palliative care, and whom the Care Manager has determined is characterized by clinical, mental, cognitive, functional, social, or behavioral complexity that necessitates the involvement of a care team to prevent health deterioration. Such a determination may be based on measurable criteria established by the Health Fund for this purpose.
- **"Administrative Care Coordinator"** – A staff member whose role is to assist the patient in performing the administrative coordination required to implement the care plan, including scheduling medical appointments, tests, and treatments, and performing necessary coordination and updates among all members of the care team and community entities.
- **"Care Team"** – A core care team or an expanded care team.
- **"Core Care Team"** – The basic care team to which a patient is assigned in primary care clinic, including personal primary care physician (who is defined as a case manager), nurse, social worker, and administrative care coordinator.
- **"Expanded Care Team"** – A care team including the core care team and at least two additional staff members, such as a secondary care physicians (e.g. cardiologists, pulmonologists, psychiatrists, pain-management physicians), psychologist, clinical pharmacist, or an allied health professional, added according to the patient’s medical or psychosocial needs.
- **"Personalized Care Plan"** – An individual plan for a complex patient that includes personal goals and steps for proactive intervention (medical or otherwise) and provides a therapeutic response for urgent medical cases. The care plan shall be determined jointly in a proactive meeting between the care team and the patient.

**B. Purpose of Support:** The purpose of support within this domain is to improve the medical care provided to complex patients by assigning them to a multidisciplinary team. This team shall operate according to teamwork principles to establish and implement a care plan in full collaboration with the patient.

**C. Threshold Conditions for Granting Support:**

1. The Health Fund (HMO) has operated a care team program aimed at improving the treatment of complex patients.
2. The program is based on the principles detailed in the Second Appendix to these criteria.
3. The program was presented to the Ministry’s Medical Division and Regulatory Division. The Ministry’s reservations were conveyed to the Health Fund and incorporated into the program.

**D. Conditions for Supported Activity:**

1. The duration of treatment for each patient shall be no less than three months, unless the duration is shorter due to circumstances beyond the Health Fund's control, including hospitalization.
2. At least one meeting must occur between each member of the **Core Care Team** and the patient within one month of their inclusion in the program, including a meeting with the **Administrative Care Coordinator**, if included in the team. Notwithstanding the above, meetings with the **Social Worker** may take place within three months of the patient's inclusion.
3. Conducted and documented medical encounters with the **Care Manager** at least once per quarter.
4. Conducted and documented encounters with a **Nurse** at least twice per quarter.
5. Conducted encounter with an **Administrative Care Coordinator**, if included in the team, at least once per quarter.
6. Conducted and documented medical encounters with each of the additional team members at least once every six months (semi-annually).
7. Establishment of a documented **Personalized Care Plan** within two months of the patient's inclusion in the program.
8. Conducting a documented professional follow-up discussion among the care team members at least once per quarter, and in any event, prior to a decision to terminate the team-based care.
9. The Health Fund has provided professional training to the care teams, in accordance with the principles established in the program, prior to the commencement of their work. For the purposes of this paragraph, team training may be conducted using digital means.

**E. Criteria for Accumulating Support Eligibility:**

1. Support shall be granted for activity periods of three treatment months completed within the evaluation year, as detailed below:
   - **a.** For treatment periods shorter than three months, in cases where treatment was terminated due to circumstances beyond the Health Fund's control, the Health Fund shall receive support proportional to the duration of the treatment, in accordance with the provisions of sub-paragraph (b) below.
   - **b.** For every three full months of treatment per patient included in the care plan and completed during the supported period, an **eligibility amount** shall be calculated for the Health Fund as follows:
     1. For a complex patient treated by an **Expanded Care Team** residing in a **geographically peripheral area**: 1,500 NIS.
     2. For a complex patient treated by a **Core Care Team** residing in a **geographically peripheral area**: 1,200 NIS.
     3. For a complex patient treated by an **Expanded Care Team** not residing in a peripheral area: 1,200 NIS.
     4. For a complex patient treated by a **Core Care Team** not residing in a peripheral area: 1,000 NIS.
     5. For a complex patient **over the age of 75**, the eligibility amount shall be multiplied by a **coefficient of 1.2**.
     6. For a patient with **psychiatric complexity**, the eligibility amount shall be multiplied by a **coefficient of 1.1**.
     7. In cases where the care team did not include an **Administrative Care Coordinator**, 200 NIS shall be deducted from the quarterly eligibility amount.
2. Should the Health Fund fail to meet the required support criteria for a specific quarter, as detailed in this subsection, it shall not accumulate eligibility for that quarter.
3. Should the Health Fund fail to perform the required semi-annual activity, it shall not accumulate eligibility for one of the quarters within that half-year.
