# Supplementary material for: Primary care staff and health policymakers’ perspectives on the Israeli special team management program for complex patients and its further enhancement: a qualitative study
Source: Isr J Health Policy Res. 2026 Nov 9;15:49. doi: 10.1186/s13584-026-00792-x (PMC13746559; doi:10.1186/s13584-026-00792-x)
Supplement: Supplementary file 3 [file 13584_2026_792_MOESM3_ESM.docx]

Interview Schedule

Interview Number ________

Interviewee ________

1. Defining the Complex Patient

Complex patients are defined as those treated within a primary care setting, who do not belong to a home-care unit and do not require palliative care, but have been characterized by their personal physician as having clinical, mental, cognitive, functional, social, or behavioral complexity, requiring the involvement of a care team to prevent health deterioration.

a. Do you believe these criteria for inclusion in the intervention program accurately define a "complex patient"? Why?

b. How would you suggest selecting patients: by the treating physician's discretion or via top-down administrative directives?

c. The Ministry of Health (MoH) support criteria refer to ages 65 and older. In your opinion, what is the appropriate age range for inclusion in the Complex Patient Program?

d. What are the five primary medical diagnoses that, in your view, characterize patients who should enter the program?

e. Assuming medical conditions are similar (e.g., two patients with Diabetes and Hypertension, similar socioeconomic status, and similar clinical balance), what are five additional diagnoses or conditions that increase the likelihood of a patient being included in the program?

f. Define up to five laboratory markers of imbalance (prolonged levels above/below critical thresholds) that can be used for proactive identification of complex patients compared to other chronic patients. Are additional markers such as CRP, Vitamin D, Albumin, or Iron important factors in this definition?

g. To what extent is over-utilization or under-utilization of visits to primary care and specialist physicians a criterion for inclusion? If so, how many visits per year are considered "over-utilization" or "under-utilization"?

h. Rate on a scale of 1 (least) to 5 (most) the extent to which the following components define a complex patient:

* Social aspects (1-5)

* Functional aspects (1-5)

* Mental health aspects (1-5)

* Cognitive aspects (1-5)

i. What additional criteria would you add or change in the definition of a complex patient?

2. The Care Plan

According to the MoH support criteria, the physician builds an individualized care plan for each patient. It is recommended that the nurse performs: a depression screening, pain assessment, malnutrition screening for the elderly, fall prevention assessment, and a functional report.

a. Do you think a general assessment should be conducted for every patient entering the program to build a personalized plan?

* If so, which tests/questionnaires/referrals/assessments would you include?

* How often should a re-evaluation be performed?

* Should the care plan be built according to uniform criteria and metrics (selectable from a database) or based on the physician’s/nurse’s personal clinical judgment?

b. Which proactive tools (up to 5) can be used in the personalized plan to promote the patient's health and improve adherence? (e.g., healthy lifestyle education, motivational interviewing, etc.).

c. On a scale of 1 to 5, how important is it to include a pharmacological treatment goal in every personalized plan for: Diabetes (disease-modifying drugs like SGLT2 and GLP1), Pain, and Depression?

* What other goals are essential to include in every plan?

Frequency of Visits

d. How often per quarter should the patient meet with:

* The Personal Physician: ______

* The Nurse: ______

* The Social Worker: ______

* The Care Coordinator: ______

* Team Meeting (Staffing): ______

e. What is the timeframe for treating a complex patient within the program? (When is the right time to discharge a patient from the program back to routine clinic follow-up?)

Outcomes

f. In your opinion, how does the program contribute to improving the health status of complex patients?

* Rate from 1 to 5 the extent to which inclusion in a managed program improves:

* ER visits (1-5)

* Hospitalizations (1-5)

* Diabetes balance (1-5)

* Functioning and treatment adherence (1-5)

* Lifestyle changes (1-5)

3. The Care Team

The core team includes a personal physician, a nurse, a social worker, and a care coordinator. Additional multidisciplinary medical staff are added as needed.

a. What is the nurse's contribution?

b. What is the personal physician's contribution?

c. What is the social worker's contribution?

d. What is the care coordinator's contribution?

e. Who among the team members is best suited to be the "Care Manager"? Why?

f. How significant is the nurse's role, and is there an advantage to the nurse leading the team?

g. Is there a need for changes in the core team? (e.g., retaining only the physician, nurse, and coordinator).

4. Future Perspectives

Which changes would you suggest to improve the program? Rate from 1 (strongly disagree) to 5 (strongly agree):

a. Dedicated clinics at the regional/district level (1-5). If yes, where should they be located and at what scale? Who should be on the staff?

b. A district-level nursing monitoring the enrolled complex patients (1-5).

c. Integration of Clinical Nurse Specialists (CNS) in complex patient management (1-5).

d. Implementation of advanced technologies (e.g., DATOS for glucose monitoring, FEEL BETTER for pharmacological consultation) to improve efficiency (1-5).

e. Dedicated virtual D2D (Doctor-to-Doctor) consultation for physicians and nurses (1-5).

f. Is there anything else you would like to add?
